# Role of voltage-dependent anion channel 1 in neurodegeneration: Mechanisms, implications, and therapeutic potential

**DOI:** 10.4103/NRR.NRR-D-25-00368

**Published:** 2025-09-03

**Authors:** Astha Parikh, Anas Cholavaram, Ajith Kumar Chitti Babu, Kanagavel Deepankumar, Murali Vijayan

**Affiliations:** 1 Department of Biotechnology, School of Biosciences and Technology, VIT University, Vellore, India; 2 Department of Internal Medicine, Texas Tech University Health Sciences Center, Lubbock, TX, USA

**Keywords:** Alzheimer’s disease, apoptosis, mitochondria, neuroinflammation, oxidative stress, Parkinson’s disease, voltage-dependent anion channel 1

## Abstract

Voltage-dependent anion channel 1 is an integral outer membrane protein of the mitochondria that governs apoptosis, enables metabolite exchange, and influences mitochondrial activity. In neurodegenerative diseases, such as amyotrophic lateral sclerosis, Parkinson’s disease, Huntington’s disease, and Alzheimer’s disease, oxidative stress, neuroinflammation, and mitochondrial dysfunction are frequent features. Voltage-dependent anion channel 1 is a key regulator of these processes. This review described the structure, membrane topology, and physiological function of voltage-dependent anion channel 1 in neurons and glial cells. We emphasize how it affects mitophagy, oxidative damage, and changes in mitochondrial permeability. Special attention is focused on how voltage-dependent anion channel 1 interacts with pathogenic proteins that damage mitochondrial integrity and cause neurotoxicity, including mutant huntingtin, phosphorylated tau, α-synuclein, amyloid-beta, and TAR DNA-binding protein 43. Furthermore, the paper examines the function of voltage-dependent anion channel 1 in astrocytic dysfunction and microglial activation, highlighting its impact on neuroinflammation. In a nutshell, we assess treatment strategies that target voltage-dependent anion channel 1, such as VBIT-4, a selective inhibitor of voltage-dependent anion channel 1 oligomerization, and newer methods, including structure-based drug design and CRISPR/Cas9 regulation. Improved knowledge of the hinter voltage-dependent anion channel 1 of the molecular mechanism may allow for new therapeutic approaches in neurodegenerative diseases.

## Introduction

Neurodegenerative diseases represent a broad spectrum of chronic and progressive disorders that primarily impact the structure and function of neurons and the overall integrity of the nervous system. Despite their diversity, these conditions commonly exhibit underlying mechanisms such as persistent neuroinflammation, the accumulation of misfolded proteins, and the gradual loss of specific neuronal populations (Cheng et al., 2021; Souder et al., 2023). Prominent neurodegenerative disorders, including Parkinson’s disease (PD), Alzheimer’s disease (AD), and amyotrophic lateral sclerosis (ALS), share hallmark features such as chronic inflammation, protein aggregation, and selective neuronal death. Aging is the primary risk factor for these conditions. Additional neurodegenerative disorders, such as tauopathies, α-synucleinopathies, amyloidoses, and TAR DNA-binding protein 43 (TDP-43) proteinopathies, further exemplify the diverse pathology of neurodegenerative diseases (Dugger and Dickson, 2017). In AD, disease pathology is characterized by hyperphosphorylated tau forming neurofibrillary tangles and extracellular amyloid-beta (Aβ) deposition in senile plaques (O’Brien and Wong, 2011; Sadigh-Eteghad et al., 2015). Conditions such as PD, encephalopathy with Lewy bodies, multiple system atrophy, pure autonomic failure, and REM sleep behavior disorder are linked to synuclein-associated neuroinflammation (Calabresi et al., 2023). Similarly, TDP-43 clustering is connected with frontotemporal dementia and ALS. These aggregates arise in distinct regions of the nervous system and progressively spread to other areas (Haque et al., 2022). While inherited genetic mutations increase susceptibility to neurodegenerative diseases, genetic resilience is also important. For example, a longevity-associated variant in the *BPIFB4* gene has been suggested to offer neuroprotection against degenerative changes (Villa et al., 2018). Studies on pathophysiological mechanisms of neurodegeneration, such as oxidative damage, have identified the NADPH oxidase 2 (NOX2) gene as a key player, along with related species such as NOX1, NOX3-5, DUOX1, and DUOX2 (Geng et al., 2020; Tu et al., 2023).

Voltage-dependent anion channel 1 (VDAC1), a beta-barrel protein encoded on human outermost cell membrane and outer mitochondrial membrane, chromosome 5 creates an ion network (Blachly-Dyson et al., 1994). It is analogous in both design and operation to its isoforms, VDAC2 and VDAC3, which regulate processes such as sperm generation, mitochondrial cell death, and respiration (Cheng et al., 2003; Alvira et al., 2012; Maldonado et al., 2013). Dysregulation of VDAC1-mediated calcium transport and pore regulation contributes to conditions such as AD, PD, and malignancies (Huang et al., 2014; Smilansky et al., 2015). Notably, Lund University research has shown that preventing VDAC1 overexpression may hinder type 2 diabetes progression (Zhang et al., 2019). VDAC1 is essential for the creation of energy and metabolic regulation by facilitating adenosine triphosphate (ATP) and adenosine diphosphate (ADP) translocation across the mitochondrial wall (Smilansky et al., 2015; Ben-Hail et al., 2016). VDAC1 plays a critical role in apoptosis by facilitating the release of cytochrome c and interacting with Bcl-2 family proteins, and also its role in calcium signaling makes VDAC1 integral to cellular and mitochondrial functions (Hu et al., 2022). It also influences autophagy, inflammation, and mitochondrial-endoplasmic reticulum communication. Its position in the outer mitochondrial membrane allows it to interact with over 100 molecules, influencing cellular and mitochondrial functions through various signaling mechanisms (Kim et al., 2019). Particularly in tumor cells, proteins such as hexokinase (HK), Bcl-2, and Bcl-xL interact with VDAC1 to promote apoptosis. It oligomerizes and facilitates the release of apoptotic factors (Shoshan-Barmatz et al., 2017b; Hu et al., 2022).

Research has shown that VDAC1 expression increases with age in human brains, potentially contributing to impaired mitochondrial function. Autophagy and mitophagy are enhanced when VDAC1 is substantially suppressed, optimizing synaptic and cognitive functions (Vijayan et al., 2022). In AD models, VDAC1 overexpression exacerbates mitochondrial dysfunction and oxidative stress, contributing to neuronal cell death through interactions with tau and Aβ. Addressing VDAC1 overexpression could mitigate cognitive decline (Manczak et al., 2013; Shteinfer‐Kuzmine et al., 2018). Small molecules such as VBIT-4, which suppress VDAC1 oligomerization, have demonstrated potential in halting brain impairment and neuronal degeneration in AD studies (Verma et al., 2022). With a focus on mitochondrial dysfunction, protein interactions, oxidative stress, and neuroinflammation, this review seeks to present a thorough summary of the function of VDAC1 in neurodegenerative disorders. By exploring its structural characteristics, physiological functions, and contribution to cellular signaling, this review underscores the significance of VDAC1 as a key player in neuronal health and pathology. These insights deepen our understanding of the molecular underpinnings of aging and neurodegenerative processes while highlighting the potential of VDAC1 as a promising therapeutic target. By addressing these mechanisms, the review seeks to pave the way for innovative strategies to mitigate neurodegeneration and improve cognitive outcomes in age-related diseases.

## Structure and Function of Voltage-Dependent Anion Channel 1

### Structural characteristics

The structure of VDAC1 in a lipid bilayer has been elucidated using advanced nuclear magnetic resonance techniques, revealing a 19-stranded β-barrel and an N-terminal α-helix, which highlight the protein’s dynamic gating behavior and its interaction with cholesterol and the Bcl2-antisense oligonucleotide G3139 (Najbauer et al., 2022). The N-terminal helix is key to voltage gating; under different voltages, it shifts positions within the β-barrel, controlling the channel’s open or closed state. β-NADH binding results in a low-conductance state without altering the channel’s structure, emphasizing how ligand interactions influence conductance rather than architecture. This conformational flexibility is implicated in mitochondrial dysfunction during neurodegeneration, where misregulated gating contributes to bioenergetic failure. High-resolution nuclear magnetic resonance and molecular dynamics simulations confirm the functional importance of these dynamic regions (Böhm et al., 2020).

**Figure 1:**
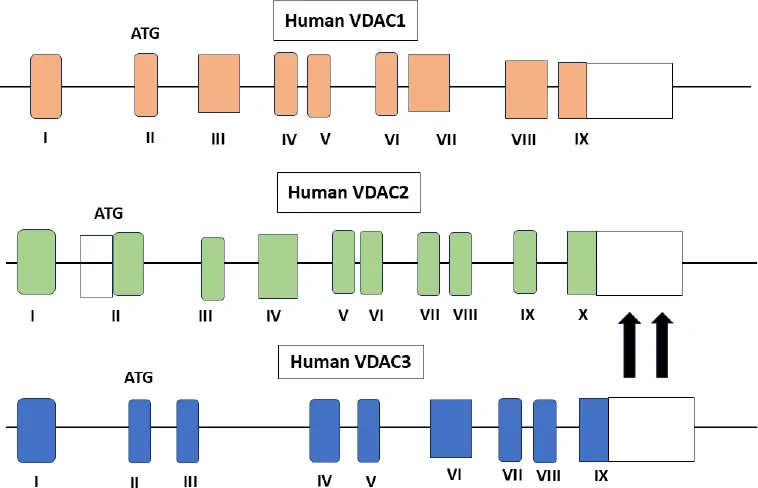
Comparison of the exon-intron structures of human voltage-dependent anion channel (VDAC) isoforms (VDAC1, VDAC2, and VDAC3). Comparative exon-intron architectures of the three human VDAC isoforms are displayed, with introns indicated as connecting lines and exons as coloured boxes. Nine exons make up the canonical structure of VDAC1 (orange). Two distinct extensions (shown by black arrows) and an extra exon (X) are present in VDAC2 (green), indicating isoform-specific regulatory involvement. Although it has nine exons as well, VDAC3 (blue) is structurally different from VDAC1. For every isoform, the translation beginning codon (ATG) is marked. These variations demonstrate the structural variety of VDAC isoforms, which could be a factor in their various functional differences in mitochondrial physiology and cellular homeostasis.

VDAC2, a related isoform, regulates mitochondrial outer membrane permeabilization through interactions with Bak and Bax, forming high-affinity complexes that facilitate apoptotic processes (Dudko et al., 2020). Isoform-specific differences in VDAC channels have been observed, including their interactions with alpha-synuclein and calcium permeability. Mutational and molecular dynamics analyses have explored the structural basis of unique plasticity of VDAC2 (Rosencrans et al., 2023). Modified peptides based on the N-terminal helix of hexokinase I enhance their interaction with VDAC, preventing peripheral nerve demyelination and providing potential therapeutic insights for peripheral neuropathies (Gautier et al., 2022). Calmodulin also modulates the gating and conductivity of VDAC1, influencing mitochondrial Ca^2+^ signaling and transport properties (Koren et al., 2022). **Figure 1** illustrates the position of the N-terminal helix in open versus closed states.

### Relevance of voltage-dependent anion channel 1 in neurodegenerative diseases and aging

VDAC1 plays a critical role in mitochondrial function and has been increasingly implicated in the pathology of neurodegenerative diseases such as AD and PD. One potential therapeutic strategy involves blocking VDAC1 oligomerization using molecules such as VBIT-4, which has shown promising effects in animal models by preserving mitochondrial integrity, lowering inflammation, and enhancing neuronal survival and cognitive performance (Verma et al., 2022). VDAC1 not only serves essential roles in apoptosis and metabolism but also emerges as a significant target in both neurodegenerative and cancer research due to its involvement in cellular energy regulation (Shoshan-Barmatz, et al., 2017a). Beyond its neurological relevance, VDAC1 has been linked to the progression of type 2 diabetes, particularly through pathways associated with mitochondrial stress and dysfunction triggered by environmental toxicants and damage to insulin-producing β-cells (Ma et al., 2024).

MicroRNA-7a-5p (miR-7a-5p) has been found to regulate the VDAC1/JNK/c-JUN signaling cascade, playing a protective role by preventing mitochondrial collapse and subsequent cell death in diabetic models (Jiao et al., 2023). VDAC1 has significant physiological roles in neurons and glial cells. Silica nanoparticles cause oxidative mitochondrial damage in neurons, which can be mitigated by inhibiting VDAC1 in SH-SY5Y cells (Koren and Ghosh, 2022). Moreover, in neonatal hypoxic-ischemic encephalopathy, elevated expression of brain-derived neurotrophic factor has been shown to support axonal repair by modulating VDAC1 and synaptosomal-associated protein Stx1b, thereby limiting ischemic neuronal injury (Xue et al., 2021). Additionally, the compound 4-phenylbutyric acid offers neuroprotective benefits in PD models, possibly by attenuating oxidative damage, reducing the formation of toxic protein aggregates, and inhibiting apoptosis, all of which may involve mechanisms regulated through VDAC1 (Tiwari et al., 2022).

## Voltage-Dependent Anion Channel 1 and Mitochondrial Dysfunction in Neurodegeneration

VDAC1 plays a central role in mediating mitochondrial dysfunction in AD and other neurodegenerative disorders through its interactions with misfolded proteins such as Aβ and hyperphosphorylated tau (Manczak and Reddy, 2012). VDAC1 interacts with misfolded proteins, such as Aβ and hyperphosphorylated tau, leading towards mitochondrial dysfunction, oxidative damage, calcium imbalance, and cell death (Shteinfer‐Kuzmine et al., 2018; Verma et al., 2022). Neurons around Aβ deposits exhibit increased VDAC1 expression, contributing to neuronal death (Verma et al., 2022). In affected neurons, mitochondrial binding of Aβ and phosphorylated tau promotes axonal degeneration and enhances oxidative stress via mitochondrial matrix proteins such as cyclophilin D (CypD) and amyloid-binding alcohol dehydrogenase. These interactions disrupt mitochondrial permeability, hinder metabolite transport, and exacerbate mitochondrial dysfunction. Elevated CypD levels further aggravate these abnormalities in AD (Reddy, 2013).

α-Synuclein plays a dual role in mitochondrial function by offering oxidative protection but also competing with mitochondrial import channels such as the TIM/TOM complex and VDAC, disrupting mitochondrial homeostasis. This antagonism affects the expression and function of mitophagy-related genes, including PINK1, PARKIN, and LRRK2 (Pozo Devoto and Falzone, 2017; Haque et al., 2022). Mitochondria, with their double biolipid membranes, are central to cellular life and death. The inner mitochondrial membrane houses nucleotide transporters and electron transport chains; however, small molecules can penetrate through the outer membrane. Peptides such as VDAC1, adenine nucleotide translocase (ANT), and CypD regulate mitochondrial permeability. Studies indicate enhanced concentrations of these proteins in AD brains after death and transgenic mouse models (Manczak et al., 2013). VDAC1 and VDAC2 establish porosity in the outer mitochondrial membrane, a function absent in recombinant VDAC3 (Wang et al., 2024). VDAC1 is sensitive to changes in transmembrane voltage; once the voltage exceeds 20–30 mV, the channel alters its conformation to regulate the transport of ATP, ADP, and other metabolites. In diseased neurons, this voltage-dependent gating is compromised, limiting ATP availability and contributing to cellular energy deficits (Vander Heiden et al., 2000; Duan et al., 2003). Apoptotic inhibitors such as Bcl2-XL suppress VDAC1 activation, while pro-apoptotic factors such as tBid stimulate pore closure, highlighting the dual role of VDAC1 in mitochondrial life and death pathways (Vander Heiden et al., 2000; Rostovtseva and Bezrukov, 2012). Cytoskeletal proteins and tau aggregates disrupt VDAC1 conductance, impairing energy metabolism. Studies on tubulin-VDAC interactions have demonstrated blocked metabolite transport and reduced mitochondrial function, further exacerbating mitochondrial dysfunction in diseases such as AD. Complexes formed by tau, VDAC1, and Aβ obstruct mitochondrial channels, impairing energy metabolism and calcium homeostasis (Rostovtseva and Bezrukov, 2012; Reddy, 2013). Calcium regulation is another key function of VDAC1, critical for ATP synthesis and cell viability. Dysregulation of VDAC1-mediated calcium transport causes mitochondrial calcium overload, leading to diminished discharge of pro-apoptotic factors, membrane potential, and permeability transition pore closure (Gincel et al., 2001; Rongvaux et al., 2014).

Mitochondrial impairment is also one of the major contributing factors in a wide range of neurodegenerative conditions, including AD, PD, and Huntington’s disease. In AD, mitochondrial failure accelerates the accumulation of Aβ and tau aggregates. PD is characterized by synaptic dysfunction and memory impairment due to oxidative damage and disrupted mitochondrial dynamics. In Huntington’s disease, expanded polyglutamine repeats in the *HTT* gene interfere with mitochondrial function, leading to neuronal apoptosis and energy imbalance (Tomasello et al., 2009; Shteinfer‐Kuzmine et al., 2018; Verma et al., 2022). Restoring mitochondrial homeostasis is a therapeutic goal across these conditions. Agents that induce mitophagy, reduce reactive oxygen species (ROS), and enhance mitochondrial biogenesis help restore mitochondrial homeostasis. It is also found that certain kinases and phosphatases regulate mitochondrial metabolic processes through protein phosphorylation and dephosphorylation. Post-translational modifications of mitochondrial proteins influence mitochondrial dynamics and neuronal health. Notably, mitophagy regulators such as Parkin and PINK1 help maintain mitochondrial integrity. Furthermore, overexpression of transport proteins such as the dynein adaptor snapin has been shown to correct lysosomal trafficking defects and improve motor neuron survival (Xie et al., 2015; Lin and Luo, 2019).

### Mitochondrial permeability transition pore development and cell death linking voltage-dependent anion channel 1

Porins, including VDACs, are critical for maintaining mitochondrial permeability and sustaining synaptic plasticity. These proteins influence mitochondrial structure, hexokinase interaction, and apoptosis signaling pathways (Shimizu et al., 1999; Raghavan et al., 2012). VDAC1 forms part of the mitochondrial permeability transition pore (mPTP), a high-conductance channel in the outer mitochondrial membrane, with contributions from ANT, PiC, and CypD. Under stress-induced high-conductance states, mPTP opening causes apoptosis, loss of membrane potential, and mitochondrial swelling. The inner mitochondrial membrane contains ANT, while the outer mitochondrial membrane includes VDAC, both of which are considered structural components of mPTP (Gutierrez-Aguilar and Baines, 2015; Jia and Du, 2021; Moya et al., 2021). VDAC1 regulates inflammation and apoptosis via its modulation of mPTP activity. The mPTP, approximately 1.4 nm in diameter, facilitates ion transport and solute exchange for molecules smaller than 1500 kDa, earning its designation as a mitochondrial macro-channel (Giorgio et al., 2018; Urbani et al., 2019). Overexpression of VDAC1 triggers mPTP formation, leading to mitochondrial dysfunction, increased permeability, and oxidative damage. However, silencing VDAC1 inhibits these processes, preventing cell death (Tomasello et al., 2009). The activity of VDAC1 is influenced by its association with pro-apoptotic Bcl-2 family proteins, impacting the destiny of cells. Dysregulated interactions between VDAC1, phosphorylated tau, and Aβ further promote mPTP release, contributing to neuronal death in AD (Smilansky et al., 2015; Bernardi et al., 2023).

### Interplay between Bcl-2 family proteins and apoptosis modulation

**Figure 2:**
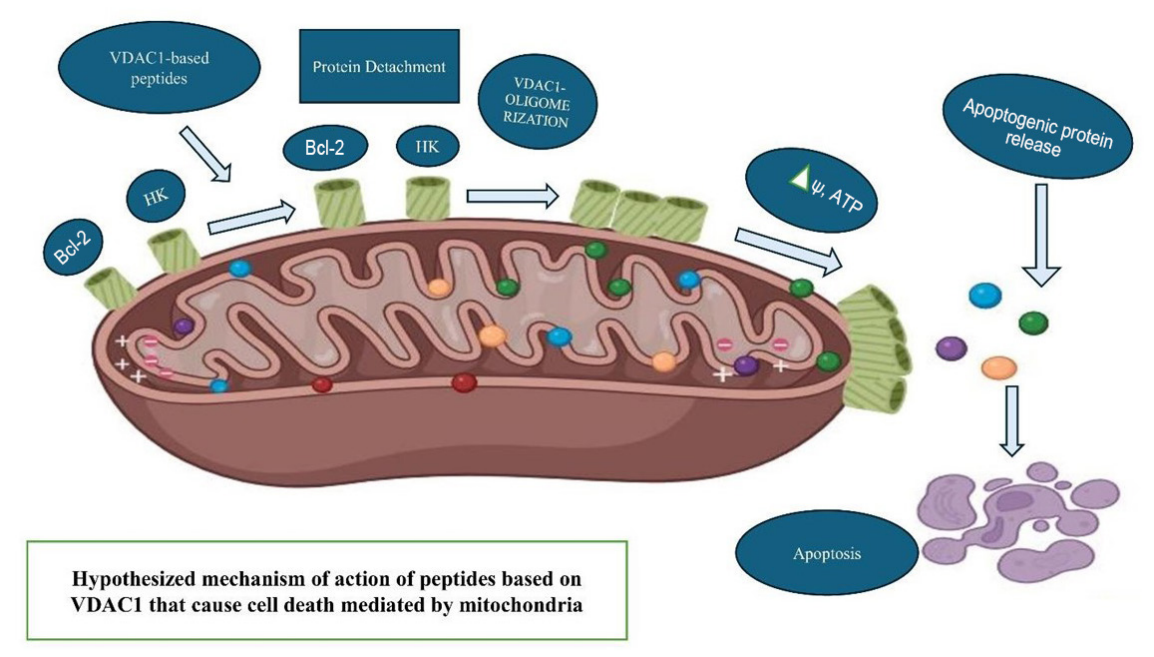
Hypothesized mechanism of voltage-dependent anion channel 1 (VDAC1)-based peptides in mitochondria-mediated apoptosis. This schematic represents the proposed mechanism by which VDAC1-based peptides induce mitochondria-mediated cell death. VDAC1, a mitochondrial outer membrane protein, plays a crucial role in regulating apoptosis by controlling metabolite flux and interactions with anti-apoptotic proteins. The figure illustrates how VDAC1-based peptides promote protein detachment, leading to the release of key mitochondrial regulators such as Bcl-2 and hexokinase (HK) from VDAC1. This event facilitates VDAC1 oligomerization, disrupting mitochondrial integrity and increasing permeability. Consequently, mitochondrial membrane potential (Ψm) decreases, resulting in adenosine triphosphate (ATP) depletion and the release of apoptogenic proteins into the cytosol. These released factors activate apoptotic signaling cascades, ultimately leading to programmed cell death. Created with BioRender.com.

The amino acid sequences of the Bcl-2 family play a key role in controlling both the health of mitochondria and cell suicide (Green and Reed, 1998). VDAC1 interacts with Bcl-2-associated X protein (BAX), a pro-apoptotic member of the Bcl-2 family, through cell suicide to coordinate the expulsion of cytochrome c (Yang et al., 1997; Shimizu et al., 1999). BAX oligomerization in the mitochondrial membrane increases pore size, enhancing mitochondrial permeability and promoting apoptosis (Banerjee and Ghosh, 2004; Peña‐Blanco and García‐Sáez, 2018; **Figure 2**). During apoptosis, VDAC1 tends to form oligomeric structures that create pores in the mitochondrial membrane, allowing cytochrome c to escape into the cytoplasm. Once released, cytochrome c interacts with apoptotic factors such as apoptotic protease-activating factor-1 and deoxyadenosine triphosphate, leading to the assembly of apoptosomes. These apoptosomes activate initiator caspase-9, which subsequently triggers a cascade involving effector caspases such as caspase-3, caspase-6, and caspase-7, ultimately resulting in programmed cell death (Green and Reed, 1998; Elmore, 2007; Wang and Youle, 2009). This cascade is central to the intrinsic apoptotic pathway. Mitochondrial outer membrane permeabilization also initiates inflammation. After mitochondrial DNA (mtDNA) escapes into the cytoplasm, pro-inflammatory signaling becomes operational and cyclic GMP-AMP synthase (cGAS)-stimulator of interferon genes (STING) networks are triggered (Sun et al., 2012; Riley et al., 2018; Huang et al., 2020). Additionally, mitochondrial outer membrane permeabilization can disrupt inhibitors of apoptosis proteins, promoting proteasomal degradation and activating caspase-8. This cascade enhances pro-inflammatory nuclear factor-κB signaling and triggers release of interleukin-1β (IL-1β) (Riley et al., 2018).

## Voltage-Dependent Anion Channel 1 and Oxidative Stress

The brain and nervous system are highly metabolically active and rely on a constant supply of oxygenated blood. Any interruption in blood flow can lead to oxidative stress, resulting in the overproduction of reactive nitrogen and oxygen species and free radicals (Nunomura et al., 2006; Shukla et al., 2011). Oxidative damage, a significant contributor to neurodegenerative diseases, is exacerbated by age-associated alterations in mtDNA, leading to psychological decline and age-related neurodegeneration (Lin and Beal, 2006). Accelerated peroxidation of fatty acids, protein oxidation, and DNA/RNA base alterations are markers for oxidative damage (Markesbery and Lovell, 2007).

Oxidative stress is a hallmark of disorders such as AD, PD, and ALS. ROS accumulation and antioxidant responses are out of balance, which leads to it. Processes such as mitochondrial dysfunction, protein oligomerization, ubiquitin-proteasome system defects, cytokine production, and blood-brain barrier disruption exacerbate oxidative damage in these conditions (Sienes Bailo et al., 2022; Olufunmilayo et al., 2023; Veselov et al., 2023). Glial cells, especially microglia and astrocytes, contribute to oxidative neuroinflammation via signaling pathways such as nuclear factor-κB, Nrf2, and STAT3, further linking oxidative stress to neurodegeneration (Lee et al., 2023). Furthermore, mitochondrial dysfunction caused by ROS overproduction compromises oxidative-reductive reactions, disrupts blood-brain barrier integrity, and induces endothelial cell necrosis, which are common in neurodegenerative diseases (Picca et al., 2020).

Oxidative stress and poor energy metabolism in the brain are the main causes of neurodegenerative illnesses, such as PD, AD, and ALS. These processes lead to neuronal cell death and deteriorating motor or cognitive functions. Neurons are particularly vulnerable due to their high fatty acid content, elevated oxygen consumption, and limited antioxidant defenses (Singh et al., 2019). ROS overproduction overwhelms antioxidant defenses, resulting in oxidative damage, cellular dysfunction, and death in neurons with inherently low antioxidative capacity (Teleanu et al., 2022). Oxidative damage is further exacerbated by an imbalance in the natural antioxidant mechanisms of the nervous system, leading to neurodegeneration and subsequent neuronal loss (Gandhi and Abramov, 2012; Houldsworth, 2023; Olufunmilayo et al., 2023).

VDAC1, as a mitochondrial membrane protein, plays a critical role in oxidative stress regulation by modulating mitochondrial permeability and facilitating the release of apoptotic factors (**Table 1**). Its involvement in ROS sensing and response makes it a key regulator of oxidative damage. VDAC1 knockout increases oxidative stress and ROS levels in certain cell types, suggesting a protective role against oxidative damage under specific conditions (Tomasello et al., 2009; Brahimi-Horn et al., 2015; Camara et al., 2017). VDAC1 forms complexes with mitochondrial creatine kinase and ANT, facilitating ATP/ADP exchange critical for maintaining cellular energy balance (Camara et al., 2017). During oxidative stress, VDAC1 oligomerizes, enhancing mitochondrial permeability and apoptotic signaling. Among the post-translational changes that impact its operation are redox-sensitive cysteine residues that undergo structural changes in response to ROS, altering the function of VDAC1 (Brahimi-Horn et al., 2015; Shoshan-Barmatz et al., 2017b).

**Table 1:** VDAC1 plays a role in oxidative stress and neuroinflammation in a variety of neurodegenerative disorders

Neurodegenerative disease	Findings on VDAC1	Oxidative stress contribution	Neuroinflammation involvement	Mitochondrial dysfunction	VDAC1 interactors	Reference
Alzheimer's disease	VDAC1 upregulated in neurons	Increased ROS production, decreased antioxidant levels	Enhances microglial activation, promotes IL-6, TNF-α release	Mitochondrial permeability transition pore opening	Amyloid-beta	Argueti-Ostrovsky et al., 2024
Parkinson's disease	VDAC1 contributes to mitochondrial stress	Increases ROS, leading to dopaminergic neuron death	Promotes nuclear factor-κB activation, leading to sustained neuroinflammation	Calcium overload, adenosine triphosphate depletion	α-Synuclein, PINK1	Houldsworth, 2023
Huntington's disease	VDAC1 overexpressed in affected neurons	Triggers lipid peroxidation, oxidative DNA damage	Enhances pro-inflammatory cytokine release	Increased mitochondrial fragmentation	Mutant Huntingtin	Gao et al., 2014
Multiple sclerosis	VDAC1 involved in oligodendrocyte stress	Promotes nitric oxide-mediated oxidative damage	Induces astrocyte reactivity, promotes blood–brain barrier dysfunction	Disrupts mitochondrial calcium	TNF-α, IL-1β	Argueti-Ostrovsky et al., 2024

IL: Interleukin; ROS: reactive oxygen species; TNF: tumor necrosis factor; VDAC1: voltage-dependent anion channel 1.

Beyond its metabolic role, VDAC1 mediates immune signaling by promoting the release of mtDNA into the cytoplasm, triggering cytokine responses and inflammation (Kim et al., 2019; Hu et al., 2022; Xian et al., 2022). Impaired mitophagy, often associated with VDAC1 dysregulation, contributes to mitochondrial dysfunction in neurodegeneration (Vijayan et al., 2022; Vijayan and Reddy, 2022). The interaction of VDAC1 with hexokinase II also influences mitochondrial ROS production. While silencing VDAC1 reduces ROS production and enhances mitochondrial performance, its overexpression can exacerbate oxidative stress. For example, H9c2 cardiac cells with VDAC1 deletion showed increased vulnerability to oxidative stress, underscoring the protective role of VDAC1 against apoptosis in specific contexts (Yang et al., 2020). Mitochondrial dynamics, including fission, fusion, and mitophagy, are essential for neuronal function and survival. Disruptions in these processes promote mitochondrial fragmentation and oxidative stress in neurodegeneration. Promoting mitophagy, reducing ROS levels, and enhancing mitochondrial biogenesis could restore mitochondrial homeostasis and improve outcomes in age-related and neurodegenerative illnesses (Huang et al., 2014; Cowan et al., 2019).

## Voltage-Dependent Anion Channel 1 in Neuroinflammation

VDAC1 contributes to both neuronal death and survival signaling through its oligomerization-dependent mechanisms, positioning it as a central regulator of brain inflammation and cell fate (de Sousa et al., 2022). Spinal cord injuries trigger apoptotic signaling pathways that contribute to inflammation and subsequent cell death. Suppressing VDAC1 oligomerization reduces apoptosis without significantly affecting the neuroinflammatory response, suggesting distinct regulatory pathways (Paschon et al., 2019). Studies using 5×FAD mouse models reveal that Aβ-induced overexpression of VDAC1 leads to mitochondrial dysfunction and neuroinflammation. Pharmacological Inhibition of VDAC1 oligomerization with the small molecule VBIT-4 mitigates apoptosis, enhances neuroprotective glial responses, and restores cognitive function in treated mice (Verma et al., 2022). The VDAC1 oligomerization turns on the NLRP3 inflammasome, promoting the formation of large pores, recruitment to mitochondria, and dissociation from hexokinase, thereby facilitating calcium influx and mitochondrial ROS production. Elevated ROS can trigger the NLRP3 inflammasome, leading to the expulsion of pro-inflammatory cytokines such as IL-1β (Zhao et al., 2022; Baik et al., 2023). In parallel, VDAC1-mediated ATP release directs microglial polarization toward either pro-inflammatory (M1) or anti-inflammatory (M2) states, significantly shaping the neuroimmune response (de Sousa et al., 2022).

In AD, VDAC1 interacts with Aβ, impairing mitochondrial function and elevating oxidative stress, a major contributor in the biology of diseases (Manczak et al., 2013). Phosphorylated tau also binds to VDAC1, further aggravating mitochondrial dysfunction and contributing to neuronal death through increased ROS levels (Smilansky et al., 2015). In VDAC1^+/−^ mice, reduced lipid peroxidation and ROS production are observed alongside improved mitochondrial function, as indicated by elevated ATP levels and cytochrome oxidase activity. However, the loss of cytochrome c impairs mitochondrial antioxidant defense, exacerbating oxidative injury (Camara et al., 2010).

**Figure 3:**
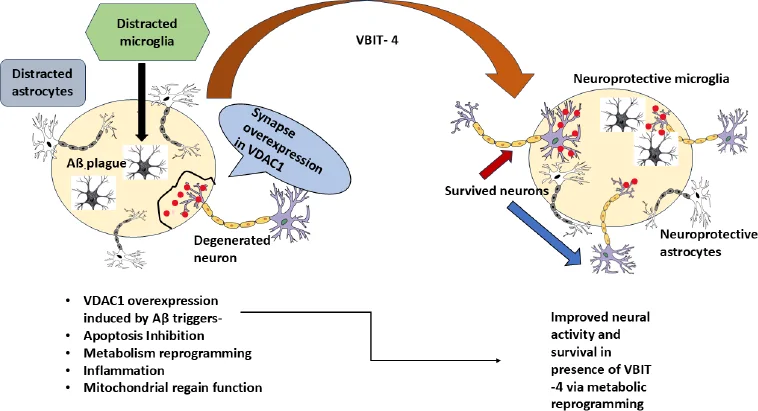
Role of VBIT-4 in counteracting voltage-dependent anion channel 1 (VDAC1) overexpression and neurodegeneration. This schematic illustrates the impact of VDAC1 overexpression on neurodegeneration and the potential neuroprotective effects of VBIT-4. Left Panel (pathological condition): amyloid-beta (Aβ) plaques trigger VDAC1 overexpression at synapses, leading to apoptotic inhibition failure, metabolic dysfunction, inflammation, and mitochondrial impairment. As a result, neurons degenerate, and microglia and astrocytes become distracted, failing to provide neuroprotection. Right Panel (therapeutic intervention with VBIT-4): Treatment with VBIT-4 restores metabolic homeostasis, reprogramming microglia and astrocytes into a neuroprotective state. This leads to neuronal survival, reduced inflammation, and improved mitochondrial function, ultimately enhancing neural activity and resilience. This figure highlights therapeutic potential of VBIT-4 in mitigating Aβ-induced neurotoxicity by targeting VDAC1-mediated metabolic dysregulation in neurodegenerative diseases.

VDAC1 regulates ATP release and influences microglial polarization, influencing neuroinflammation and neurodegenerative disorders. By triggering the NLRP3 inflammasome, VDAC1 amplifies mitochondrial ROS production, driving inflammatory responses (Camara et al., 2017). Dissociation of VDAC1 from hexokinase shifts microglial metabolism from oxidative phosphorylation to glycolysis, promoting pro-inflammatory phenotypes (M1) (Zhang et al., 2019). Dysregulated calcium signaling further exacerbates neuroinflammatory pathways, with microglia playing a pivotal role in Aβ clearance and the neuroinflammatory reflex (Huang et al., 2024). Studies demonstrate VDAC1 overexpression around neuronal terminals near Aβ plaques. VBIT-4 treatment protects microglia by preventing structural damage and restoring anti-inflammatory responses (Verma et al., 2022; **Figure 3**). Additionally, inhibition of VDAC1 affects TSPO expression and reduces microglial overactivation, contributing to neuroprotection (de Sousa et al., 2022).

Main immune cells of the brain and spinal cord, microglial cells and astrocytes, exhibit morphological and functional changes during activation. Microglial activation is marked by increased surface markers such as CD68 and IBA1 (Hopperton et al., 2018), while astrocytes undergo reactive astrogliosis, shifting from neuroprotective to pro-inflammatory phenotypes, exacerbating neuroinflammation (Novakovic et al., 2023). Astrocyte-derived CCL2 plays a crucial role in immune cell recruitment and chronic glial activation (Kim et al., 2014).

Neurodegenerative disorders often induce cross-talk between pro-inflammatory microglia and astrocytes. Aβ and phosphorylated tau activate microglia and astrocytes, releasing inflammatory mediators such as IL-1β, tumor necrosis factor-α, and IL-6 (Verma et al., 2022). Conversely, cytokines such as IL-4, IL-13, and IL-10 induce anti-inflammatory (A2) astrocytes and M2 microglia, promoting repair and resolution (Kwon and Koh, 2020). Targeting VDAC1 reorganizes inflammatory responses and reduces oxidative stress-induced cell death (de Sousa et al., 2022). Accumulated Aβ plaques stimulate microglial and astrocytic cytokine release, amplifying Aβ aggregation and inflammation (Giovannoni and Quintana, 2020). VBIT-4 restores calcium homeostasis, promotes Aβ clearance, and suppresses IL-1β production by regulating NLRP3 (Shimada et al., 2012; Ben-Hail et al., 2016). Together, these findings underscore VDAC1 as a viable therapeutic target to suppress chronic inflammation and preserve neural integrity.

## Voltage-Dependent Anion Channel 1 and Astrocytic Function

VDAC1 plays a fundamental role in astrocyte physiology, particularly in signal transduction and cellular metabolism. It is integral to maintaining calcium balance within astrocytes, but when overexpressed, it can lead to calcium overload, contributing to inflammatory responses within the central nervous system. In neurodegenerative diseases such as AD, VDAC1 has been shown to drive a metabolic transition in astrocytes from oxidative phosphorylation to a more glycolytic state, an adaptation often linked to disease progression (Smilansky et al., 2015; Shoshan-Barmatz et al., 2017b). Its presence in the astrocyte plasma membrane allows for tight control of ion and metabolite transport, which supports the firing of neurons and preserves cellular homeostasis. ROS generated by astrocytes also affect inflammatory responses and neurodegeneration, further underscoring the pivotal role of VDAC1 in brain health. Dysregulation of VDAC1 compromises the neuroprotective capabilities of astrocytes (Verma et al., 2022).

In 5×FAD animal models of AD, the VDAC1 oligomerization inhibitor VBIT-4 has been shown to restore diminished enzymatic activities such as citrate synthase and cytochrome c oxidase, thereby recovering astrocytic physiological functions (Verma et al., 2022). In addition to it, astrocytes are critical to neuronal support, contributing by shuttling metabolic substrates, releasing growth factors, and regulating pH and sodium levels within the brain microenvironment (Oakley et al., 2006). VDAC1’s modulation of calcium flux may also affect gliotransmitter release, influencing synaptic plasticity and astrocyte-neuron communication. Reactive astrogliosis, marked by astrocyte proliferation and inflammatory cytokine production, is a hallmark of AD pathology observed in both patient tissues and experimental models (Rodríguez-Arellano et al., 2016).

### Influence on astrocytic support of neurons

Beyond metabolic regulation, VDAC1 indirectly influences key astrocytic functions that support neuronal activity. Astrocytes play a fundamental role in maintaining central nervous system homeostasis by recycling neurotransmitters such as glutamate, crucial for preventing excitotoxicity (Ricci et al., 2009). Additionally, astrocytes regulate extracellular ion concentrations, including potassium, to preserve the ionic balance required for neuronal excitability (Benarroch, 2005). Astrocytes also provide lactate as an energy source for active neurons (Pellerin and Magistretti, 1994). Furthermore, they release gliotransmitters that modulate synaptic activity, strength, and plasticity (Araque et al., 1999). However, during pathological conditions such as neuroinflammation, astrocytes can become reactive, resulting in the production of cytokines that promote inflammation. If left unchecked, these cytokines may cause neuronal injury (Giovannoni and Quintana, 2020).

## Voltage-Dependent Anion Channel 1 in Aging

VDAC1 significantly impacts aging by regulating mitochondrial dysfunction, a primary contributor to age-related diseases and cellular degeneration. TRIM31 regulates dopaminergic neuron homeostasis in PD by promoting VDAC1 degradation, presenting a potential therapeutic target (Feng et al., 2024). Additionally, VDAC1 expression in neuronal cells is regulated by the AMP-activated protein kinase/peroxisome proliferator-activated receptor gamma coactivator 1-alpha (PGC-1α) and p53 pathways, which respond to metabolic and oxidative stress, influencing stress adaptation and apoptosis (Wang et al., 2024). Upregulating VDAC1 in ALS models has been shown to improve mitochondrial function by stabilizing the Complex I-Sirt3 axis, offering potential therapeutic benefits (Magrì et al., 2024).

Building on these insights, other regulators such as TMBIM6 interact with VDAC1 to preserve mitochondrial Ca^2+^ balance and enhance cardiac function, as demonstrated in models of septic cardiomyopathy (Zhou et al., 2023). Similarly, Resveratrol, a natural compound, protects against PD by modulating VDAC1 to improve mitochondrial function and reduce α-synuclein accumulation (Feng et al., 2024). Furthermore, the *UBA52* gene regulates mitochondrial homeostasis and protects neurons from PD-related cell death by modulating VDAC1 function (Tiwari et al., 2023).

Targeting VDAC1 in AD has also shown promise in restoring mitochondrial function, offering a potential approach to halting disease progression (Aran and Singh, 2023). Parkin regulates endoplasmic reticulum (ER) and power house of the cell contact sites by modulating mono- and poly-ubiquitination of proteins such as VDAC1, thereby affecting calcium homeostasis (Pereira et al., 2023). Mitophagy, crucial for neuronal health, is associated with senescence and neurological conditions such as AD and PD. Impairment of this process exacerbates mitochondrial dysfunction (Banarase et al., 2023). The transcriptional co-activator PGC-1α, which regulates neuronal metabolism, responds to aging and glycogen synthase kinase-3 beta inhibition. Lithium alters PGC-1α activity, impacting mitochondrial function, growth signaling, and gene expression, underscoring its role in brain aging (Souder et al., 2023).

VDAC1 overexpression is closely linked to mitochondrial dysfunction in various diseases, with inhibitors such as VBIT-4 demonstrating therapeutic potential (Rinaldi, 2023). Age-related decreases in mitochondrial function, specifically in ATP5F1A and TFAM, contribute to skin aging by impairing energy metabolism and epidermal renewal (Vidali et al., 2023). Mitochondrial fragmentation and elongation activate inflammatory pathways through mtDNA mislocation, involving VDAC pores, TLR9, and cGAS. These mechanisms highlight the complex roles of mitochondrial dynamics in inflammation (Irazoki et al., 2023). MIRO-1 interacts with VDAC1 to preserve the potential of the outer layer of the mitochondria and activity in fragmented mitochondria (Vidali et al., 2023). However, VDAC1 knockout in human cells disrupts mitochondrial respiration and alters electron transport chain enzyme activity (Magrì et al., 2023). Furthermore, VDAC1 oligomerization and its interaction with MLKL during ischemia-reperfusion injury increase mitochondrial membrane permeability and exacerbate cell death (Wan et al., 2024).

## Role of Voltage-Dependent Anion Channel 1 in Cellular Senescence

VDAC1 is pivotal in cellular stress responses, modulating the output of factors that cause apoptosis from mitochondria and interacting with Bcl-2 and hexokinase to balance cell survival and death pathways (Aran and Singh, 2023). In hepatocellular carcinoma, drugs such as metformin disrupt the VDAC1 complex to induce autophagy and inhibit tumor cell proliferation, suggesting the relevance of VDAC1 in cancer therapy (Ko et al., 2024). Additionally, HKDC1, a transcriptional target of transcription factor EB (TFEB), is critical in coordinating PINK1/Parkin-dependent mitophagy and lysosomal repair, maintaining mitochondrial–lysosomal balance during senescence (Cui et al., 2024).

VDAC1 has also emerged as a key biomarker in neurodegenerative aging, with studies identifying six genes, FUNDC1, MAP1LC3A, CSNK2A1, VDAC1, CSNK2B, and ATG5, which have been identified as biomarkers for AD, aiding in subtype classification and personalized treatment approaches (Ma et al., 2023). Additionally, VDAC1 facilitates PHB2 exposure during mitophagy by enhancing its interaction with LC3, which is essential for mitochondrial quality control (Roy et al., 2023). This interaction underscores the importance of VDAC1 in mitochondrial quality control and aging-linked neurodegeneration.

## Voltage-Dependent Anion Channel 1 Interactions with Neurodegenerative Disease-Associated Proteins

Mitochondria-associated membranes are dynamic regions connecting mitochondria and the ER, playing significant roles in cellular functions. Disruption of MAM-associated proteins has been linked to conditions including neurodegeneration, type 2 diabetes, and non-alcoholic fatty liver disease (Mao et al., 2022). Neurodegenerative diseases involve mitochondrial dysfunction caused by clustered proteins such as Aβ, tau, α-synuclein, and mutant huntingtin. These aggregates impair energy metabolism, promote oxidative stress, and contribute to neurotoxicity (Abramov et al., 2017). These pathological proteins often mimic mitochondrial targeting sequences, interfering with normal mitochondrial import machinery and triggering neurotoxic cascades (Norat et al., 2020). Additionally, disruptions in membrane contact sites near ER, mitochondria, and lysosomes contribute to early neurodegenerative processes in AD and PD (Vrijsen et al., 2022). In diseases such as PD, alterations in PRKN and PINK1 impair mitochondrial health, leading to neuronal death, while other proteins such as TDP-43, α-synuclein, and Miro1 also contribute to neurodegeneration (Panda et al., 2023).

### Protein-protein interaction in voltage-dependent anion channel 1

Mitochondria play a pivotal role in neurodegenerative disorders through altered protein interactions. Addressing these interactions could improve diagnostic and therapeutic strategies (Zilocchi et al., 2020). In spinocerebellar ataxia type 3, impaired Parkin–VDAC1-mediated mitophagy disrupts mitochondrial quality control (Harmuth et al., 2022). Abnormal fission, fusion, and mitophagy processes-largely mediated by Drp1-further affect neuronal survival. Interactions between Drp1 and PD-related proteins, such as α-synuclein and Parkin, influence mitochondrial dynamics, exacerbating neurodegeneration (Feng et al., 2020). Large-scale proteomic analyses have uncovered over 1900 VDAC1-related interactions, many of which are implicated in neurodegenerative disease mechanisms (Malty et al., 2017).

### Parkinson’s disease and α-synuclein

PINK1 and Parkin modulate mtDNA quality assurance by targeting VDAC1 for ubiquitination via mitophagy. Mutations in Parkin inhibit this pathway, promoting mitochondrial dysfunction in PD (Geisler et al., 2010). Enhancing the PINK1/Parkin pathway has shown potential for restoring mitophagy and improving mitochondrial health in PD (Khalil et al., 2015). During oxidative stress in PD, the DJ-1 protein translocates to power house of the cell in a VDAC1-dependent manner, enhancing ATP production and promoting chances of the cell to survive (Hewitt, 2016). DJ-1 also maintains ER-mitochondria associations via interactions with the IP3R3-Grp75-VDAC1 complex. Its deficiency disrupts mitochondrial function and calcium signaling, contributing to PD pathogenesis (Liu et al., 2019). Moreover, α-synuclein disrupts contact areas between the ER and mitochondria, affecting calcium equilibrium and mitochondrial bioenergetics. The extent of this disruption is dosage-sensitive, with both overexpression and underexpression of α-synuclein harming mitochondrial physiology (Erustes et al., 2022). ALS involves interactions with superoxide dismutase 1 (SOD1) and TDP-43. In ALS, mutant SOD1 proteins gain toxic functions, causing protein misfolding and abnormal interactions that disrupt motor neuron function. Understanding these conformational changes may help identify potential diagnostic and therapeutic targets (Huai and Zhang, 2019).

### Voltage-dependent anion channel 1 and mutant huntingtin protein

Mutant huntingtin disrupts mitophagy by impairing autophagosomes’ focusing on impaired mitochondria. Overexpression of PINK1 has been shown to partially rescue mitochondrial function in Huntington’s models (Khalil et al., 2015). Conversely, mutant huntingtin promotes the breakdown of the external membrane protein of the mitochondria MCL1 via the VCP-UBXD1 complex, contributing to mitochondrial dysfunction (Guo and Qi, 2017). Its interaction with Drp1 also disrupts mitochondrial fission, axonal transport, and synaptic homeostasis (Shirendeb et al., 2012).

## Interaction with Amyloid-Beta and Tau Proteins

**Figure 4:**
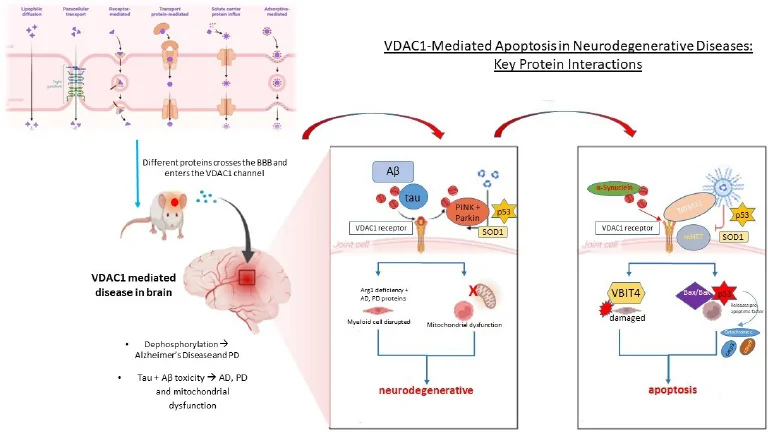
VDAC1-mediated apoptosis in neurodegenerative diseases: Key protein interactions. This schematic illustrates the role of VDAC1 in neurodegeneration, highlighting key protein interactions that drive apoptotic pathways in AD, PD, and related disorders. Proteins such as Aβ, tau, PINK1, Parkin, and p53 interact with VDAC1, leading to mitochondrial dysfunction, oxidative stress, and myeloid cell activation, ultimately triggering neuronal apoptosis and disease progression. VBIT-4, a proposed VDAC1 inhibitor, counteracts apoptotic signaling, restoring mitochondrial integrity and reducing oxidative damage, thereby preventing neuronal cell death. This figure highlights the critical role of VDAC1 in mitochondrial-mediated apoptosis and the potential for VDAC1-targeted therapeutic strategies in neurodegenerative disease management. Created with BioRender.com. AD: Alzheimer’s disease; Aβ: amyloid-beta; mHTT: mutant huntingtin; PD: Parkinson’s disease; SOD1: superoxide dismutase 1; VDAC1: voltage-dependent anion-selective channel 1.

Aβ interacts with VDAC1 in neuronal lipid rafts, leading to its dephosphorylation and promoting cell death, suggesting a significant role in AD progression (Fernandez-Echevarria et al., 2014; **Figure 4**). VDAC1 also interacts with phosphorylated tau, exacerbating malfunction of the mitochondria. Reducing the levels of VDAC1, Aβ, and tau has been demonstrated to repair the functioning of mitochondria and improve cognitive outcomes in AD models (Manczak and Reddy, 2012). Additionally, deficiencies in Arg1 in brain myeloid cells disrupt immune responses, exacerbating Aβ pathology. Proper Arg1 function is crucial for microglial activity and immune balance. Proper Arg1 function is crucial for microglial activity and immune balance (Ma et al., 2021). Moreover, Aβ impairs the activation of voltage-gated sodium channels in inhibitory neurons, leading to abnormal action potentials and network dysfunction in AD. These findings underscore the need for revised models to better capture electrophysiological changes in AD neurons (Perez and Ullah, 2018).

## Voltage-Dependent Anion Channel 1 and Mitophagy: Molecular Mechanisms

VDAC1, a protein located on the outer mitochondrial membrane, is essential for energy production, mitophagy regulation, oxidative stress mitigation, and Ca^2+^ transport (Onishi et al., 2021). Abnormalities in VDAC1 are linked to impaired mitochondrial function, inflammation, and a range of chronic diseases. Maintaining mitochondrial integrity necessitates mitophagy, which is the targeted destruction of damaged mitochondria. When this mechanism is disturbed, heart failure, cancer, and neurological illnesses are implicated (Onishi et al., 2021). VDAC1’s regulation of apoptosis and Ca²⁺ signaling makes it a promising therapeutic target for inflammation-related disorders (Hu et al., 2022). Key proteins, including Parkin and PINK1, are central to mitophagy and play vital roles in cellular maintenance and health (Valente et al., 2004).

PD has been closely associated with Parkin, an E3 ubiquitin ligase encoded by the *PARK2* gene, and PINK1, a kinase encoded by the PARK6 (Valente et al., 2004). In the mitophagy signaling pathway, several proteins work in tandem. PINK1 detects mitochondrial damage and activates Parkin to amplify the signal through ubiquitin chains. Deubiquitinating enzymes modulate the activity of Parkin and its mitochondrial targets (Nezich et al., 2015). Parkin also aids in lysosome biogenesis via nuclear translocation of TFEB (Pirooznia et al., 2022). In healthy mitochondria, PINK1 is brought into robust mitochondria by TOM complexes, where it is degraded (Wang et al., 2020). Upon mitochondrial damage, PINK1 stabilizes on the outer membrane, undergoes autophosphorylation, and activates Parkin, initiating mitophagy (Greene et al., 2012).

Ubiquitination by activated Parkin proteins such as MFN1, MFN2, and VDAC1, amplifies the ubiquitination signal to ensure selective degradation of damaged mitochondria (Geisler et al., 2010; Birsa et al., 2014). TFEB, a transcription factor regulated by Parkin and autophagy-related (ATG) proteins, enhances mitophagy by promoting Parkin activity (Kim et al., 2018). TFEB also regulates Parkin levels, which are crucial for initiating mitophagy. Deubiquitinating enzymes, such as USP30, remove ubiquitin from Parkin and other mitochondrial proteins, counteracting mitophagy. Reducing USP30 enhances mitochondrial breakdown. Other deubiquitinating enzymes, including USP8, USP15, USP33, and USP36, also influence mitophagy, though their specific roles require further investigation (Geisler et al., 2010; Cornelissen et al., 2014; Niu et al., 2020). Autophagy components such as ATG3, ATG5, and ATG7 are essential for Parkin-mediated mitophagy. Inhibiting these components disrupts mitochondrial degradation triggered by depolarization agents such as CCCP. The PINK1/Parkin pathway may involve p62/SQSTM1, which recruits damaged mitochondria to autophagosomes (Narendra et al., 2010). Recruitment of damaged mitochondria to autophagosomes may involve p62/SQSTM1 and Ambra1, which activate the Beclin-1 PI3K complex (Narendra et al., 2010). SMURF1, another E3 ubiquitin ligase, facilitates autophagosome entry of mitochondria, while Parkin promotes mitochondrial biogenesis by degrading PARIS and releasing PGC1α (Ashrafi and Schwarz, 2013).

## Voltage-Dependent Anion Channel 1 Regulation During Mitophagy

VDAC1 enables the ubiquitination of damaged mitochondria through its interaction with PINK1 and Parkin (Matsuda et al., 2010; Vives-Bauza et al., 2010). This process reduces inflammatory cytokines such as IL-1β and IL-18, which are critical for controlling inflammation. Disrupted mitophagy leads to mtDNA dispersion into the cytoplasm, activating inflammatory pathways such as TLR9 and cGAS-STING. This can result in conditions such as AD, PD, systemic lupus erythematosus, and inflammatory arthritis (Geisler et al., 2010). Targeting the PINK1/Parkin pathway, particularly VDAC1, offers promising therapeutic avenues for treating inflammation-related and autoimmune disorders (Sasaki et al., 2012; Sliter et al., 2018).

VDAC1 undergoes post-translational modifications that affect its placement, operation, and sustainability. Acetylation, including reversible lysine acetylation and irreversible N-terminal acetylation, plays a critical regulatory role (Polevoda and Sherman, 2003). Unlike VDAC2 and VDAC3, VDAC1 is subject to N-terminal acetylation following methionine removal, a process vital for its function. Lysine acetylation occurs across all isoforms, though VDAC3 acetylation is observed only under nutrient-deprived conditions (Distler et al., 2006). Understanding these post-translational modifications is essential for elucidating precise roles of VDAC1 in mitochondrial quality control (Starai et al., 2002; Kerner et al., 2012).

## Voltage-Dependent Anion Channel 1 Therapeutic Targeting

VDAC, particularly VDAC1, plays a pivotal role in apoptotic signaling, redox control, and cellular metabolism, making it a promising target for therapeutic interventions. Recognizing the effects of post-translational changes on the function of VDAC1 could open avenues for developing treatments for conditions such as cancer, neurodegeneration, and heart disease. Research into the structure and function of VDAC1, including its potential presence in the plasma membrane, could further advance therapeutic strategies (Taylor and Turnbull, 2005). Mitochondria-mediated apoptosis is critical in conditions such as heart attacks, strokes, cancer, and neurodegenerative diseases (Taylor and Turnbull, 2005). VDAC, especially VDAC1, interacts with proteins that promote and inhibit apoptosis, positioning it as a key therapeutic target for regulating apoptosis (Yagoda et al., 2007). Overexpression of VDAC1 in cancer cells highlights its role in mitochondrial protection against apoptosis. VDAC1 accumulates in brain plaques and contributes to Aβ toxicity in the case of AD (Yang and Stockwell, 2008). Variations in VDAC expression have also been observed in conditions such as Down’s syndrome, ALS, and epilepsy, linking VDAC to disease pathogenesis (Zaid et al., 2005).

VDAC1 oligomerization is a key step in initiating mitochondrial-driven apoptosis, as it facilitates the release of cytochrome c. Compounds such as VBIT-4 can block this oligomerization, offering neuroprotective effects in experimental models of AD and PD (Verma et al., 2022). Additionally, the synthetic peptides based on VDAC1 sequences have demonstrated potential in disrupting its association with pro-apoptotic proteins such as Bcl-2 and hexokinase, thereby reducing mitochondrial membrane permeabilization and limiting cell death (Gautier et al., 2022).

Cancer bioenergetics often relies on both aerobic glycolysis and oxidative phosphorylation. The Warburg effect, where tumors produce excess lactic acid even under oxygen-rich conditions, promotes tumor growth by providing metabolic intermediates for biomass synthesis (Mathupala et al., 2010). The role of mitochondria in ATP production varies by tumor type, influencing chemotherapy resistance and suggesting mitochondria as viable targets for anti-cancer therapies (Guppy et al., 2002). VDAC1, with its recently discovered NADH-binding pocket, presents a novel therapeutic target. For example, the compound SC18 disrupts VDAC function, leading to mitochondrial dysfunction and reduced cancer cell proliferation (Heslop et al., 2022).

Longevity and mitochondrial dysfunction are interrelated along with inflammation, oxidative stress, and chronic diseases (Missiroli et al., 2020). VDAC1, a key mitochondrial protein, regulates apoptosis, metabolite transport, and cellular stress responses. It also influences inflammation, mitophagy, lipid metabolism, and energy production, making it a promising therapeutic target. Recent findings emphasize the role of VDAC1 in apoptosis-related signaling and its overexpression in diseases such as ulcerative colitis (Thinnes, 2014). Additionally, VDAC1 is implicated in diabetes, cancer, and neurodegenerative diseases (Sasaki et al., 2012). High-resolution studies have identified critical regions of VDAC1 essential for its function, though to completely comprehend its regulation, further investigation has to be done. Clinical trials are exploring VDAC1-targeted treatments, such as VDA-1102, which affects glycolysis and mitochondrial activity. Early results show promise, with no significant side effects reported (Shoshan-Barmatz et al., 2010; Sasaki et al., 2012).

Mitochondrial dysfunction links neurodegenerative diseases and cancer, though their cellular impacts differ (Magrì et al., 2018). Cancer cells exhibit high glycolytic activity and resist apoptosis, whereas increased apoptosis is an identifiable feature of neurodegenerative disorders and mitochondrial damage (Shoshan-Barmatz et al., 2020). VDAC is directly linked to cell death and metabolism, potentially bridging these conditions. Located in the exterior membrane of mitochondria, VDAC controls ion and metabolite flow and engages with apoptotic regulators. Targeting VDAC in neurodegenerative diseases has proven challenging due to undefined binding sites, though compounds, peptides, and microRNAs show promise in modulating its activity (Keinan et al., 2010). In cancer, VDAC1 supports rapid cell proliferation and apoptosis inhibition, while in neurodegenerative diseases, its overexpression and hyperphosphorylation are linked to early neuronal loss (Magrì et al., 2018). Current research focuses on modifying VDAC1’s function or disrupting harmful protein interactions, though effective delivery methods remain a challenge.

VDAC1, is critical for cellular metabolism and apoptosis. Overexpression of VDAC1 is seen in AD in post-mortem brain tissues, where it interacts with Aβ, impairing mitochondrial function and leading to apoptosis and cognitive decline (Hardy, 2006; LaFerla et al., 2007). Silencing VDAC1 with specific siRNA mitigates Aβ toxicity, highlighting its possible use as an objective for therapy. The regulatory function of VDAC1 in apoptosis and metabolite transport links it to both cancer and neurodegenerative diseases (Cunnane et al., 2011). For cancer treatment, peptides and small molecules targeting VDAC1 interactions, particularly with HK, have shown potential (Herrera et al., 2011). Inhibitors that block Aβ-VDAC1 interactions are being developed for neurodegenerative disorders such as AD. Future research should focus on identifying precise binding sites and regulatory mechanisms of VDAC1, which could lead to targeted therapies (Manczak and Reddy, 2012). Combining VDAC1 inhibitors with other treatments may enhance efficacy, while identifying biomarkers associated with VDAC1 activity could improve disease monitoring and diagnosis (Haass and Selkoe, 2007).

## Research Gaps and Future Directions

VDAC1 is a key player in metabolite exchange between the mitochondria and cytoplasm, along with preserving the equilibrium of mitochondrial fuel (Shoshan-Barmatz et al., 2020). While its involvement in mitochondrial permeability and apoptosis is well-established, several questions remain unanswered, particularly regarding the precise function of pro-apoptotic protein activation of VDAC in this process. Additionally, its interactions with HK and potential therapeutic applications for regulating cell death and survival warrant further exploration (Shoshan-Barmatz et al., 2010). Mammals have evolved strict regulation of oxygen homeostasis to prevent oxidative stress from excess oxygen and ATP depletion from anoxia. The mPTP, critical in this regulation, is generated by the F1FO-ATP synthase in mammals. However, in the anoxic brine shrimp (Artemia franciscana), mPTP opening is Ca^2+^-insensitive. Comparative studies using electrophysiology, cryo-electron microscopy (cryo-EM), and fluorescence spectroscopy have shown that ATP synthase of Artemia demonstrates short, Ca^2+^-insensitive channel openings, facilitated by a unique membrane subunit structure. These findings could inform drug development for conditions such as ischemia and neurodegeneration (Kumar et al., 2024).

The VDAC channel, a beta-barrel protein on the outer mitochondrial membrane, is essential for cellular metabolism by transporting metabolites such as pyruvate, ATP, and ADP. Of the three mammalian VDAC isoforms, studies using CRISPR/Cas9 knockouts (KO) have revealed distinct physiological roles. VDAC1 KO results in minor bioenergetic deficits, VDAC3 KO leads to male sterility, and VDAC2 KO causes developmental issues. Experiments on HeLa cells show that while VDAC2 KO exhibits no significant changes, VDAC1 and VDAC3 KOs reduce mitochondrial respiration. VDAC3 KO additionally impairs spare respiratory capacity and alters metabolite consumption, emphasizing isoform-specific differences in metabolism and calcium signaling (Rajendran et al., 2023). A genome-wide CRISPR/Cas9 library screen using Mcl1-deficient mouse embryonic fibroblasts identified new regulators of apoptosis. BAX/BAK-dependent apoptosis was induced in these mouse embryonic fibroblasts by blocking pro-survival proteins (e.g., BCL-2, BCL-XL) with the BH3-mimetic ABT-737. The screen revealed that BAX interacts with VDAC2 to mediate apoptosis. sgRNAs targeting *Bak* and *Bax* genes were enriched in cells that survived ABT-737 treatment, confirming their roles in apoptosis regulation (Chin et al., 2018).

Voltage-VDAC has been identified as a critical mediator at the intersection of metabolism and cell death pathways. Recent research highlights its role in AD, where misfolded proteins such as phosphorylated tau (P-tau) and Aβ interact with VDAC1. These interactions disrupt mitochondrial function, contributing to disease progression. Postmortem AD brain analyses and animal models show that increased VDAC1 expression correlates with disease severity (Fernandez-Echevarria et al., 2014). VDAC1 binds both monomeric and oligomeric forms of Aβ, blocking mitochondrial pores and disrupting the transport of vital molecules. This disturbance triggers a cascade of cellular damage and mitochondrial dysfunction. Further evidence indicates that VDAC1 also mediates the detrimental effects of P-tau by altering channel conductance, leading to metabolite imbalance and neuronal energy deficiencies. Reducing VDAC1 expression in experimental models improved mitochondrial function, reduced neuronal damage, and enhanced cognitive outcomes (Fernandez-Echevarria et al., 2014). AD pathology also impacts the interaction between VDAC1 and HK-1, a key enzyme in glucose metabolism. In healthy mitochondria, HK-1 attaches to VDAC1 to regulate ATP production. In AD, this interaction is disrupted, leading to metabolic dysregulation and cell death. Although HK-1 inhibits VDAC1 activity during early apoptosis, it fails to maintain this regulation in advanced stages, exacerbating cell death (Magrì et al., 2018). Targeting the complex interplay among Aβ, P-tau, VDAC1, and HK-1 may provide novel therapeutic strategies for restoring mitochondrial function and mitigating AD-related neurodegeneration (Azoulay-Zohar et al., 2004; Shteinfer‐Kuzmine et al., 2018). These findings underscore the multifaceted roles of VDAC isoforms in cellular metabolism, apoptosis, and neurodegeneration. Advances in techniques such as cryo-EM and CRISPR/Cas9 have provided new insights into the structural and functional dynamics of VDAC. Future research should focus on elucidating isoform-specific roles, interactions with apoptotic regulators, and therapeutic potential in targeting VDAC1 for neurodegenerative diseases such as AD.

## Conclusion

VDAC1 serves as a critical nexus in the interplay between mitochondrial dysfunction and neurodegeneration. Its interactions with neurotoxic proteins such as Aβ and phosphorylated tau underscore its role in exacerbating mitochondrial permeability, oxidative stress, causes the loss of neurons in neurodegenerative illnesses such as AD. Advances in structural biology and gene-editing technologies have illuminated the isoform-specific roles of VDAC, offering a roadmap for precision-targeted therapies. Therapeutic strategies aimed at modulating VDAC1 expression, inhibiting its oligomerization, or restoring its functional balance show promise in mitigating neuronal damage and cognitive decline. Breakthrough treatments could be made possible by addressing VDAC1 as a goal for therapy that addresses the underlying mitochondrial pathologies in neurodegenerative diseases. Clarifying its regulation processes should be the foremost objective of additional studies. Clarifying its regulation processes should be the foremost focus of additional studies and developing clinically translatable interventions to improve outcomes in age-related neurological disorders.

## Methodology and Literature Mining

We performed a comprehensive literature search using PubMed and Scopus databases between January 2010 and March 2025, but only a few papers were considered older due to the presence of invaluable information. In the literature database, we have used certain Keywords included “VDAC1,” “neurodegeneration,” “mitochondria,” “Alzheimer’s disease,” “Parkinson’s disease,” “oxidative stress,” and “mitophagy” to obtain relevant research and review papers; in addition to this we have opted a filter where we took only English-language articles and considered them. Additional studies were identified to gather more information by manual screening and simple Google search, PubMed search, papers found via reference lists in review papers and some of the research papers.

## Data Availability

*Not applicable.*
